# Effective treatment of metastatic sentinel lymph nodes by dual-targeting melittin nanoparticles

**DOI:** 10.1186/s12951-023-02026-7

**Published:** 2023-08-01

**Authors:** Yanfeng Dai, Xiang Yu, Yuehong Leng, Xingzhou Peng, Junjie Wang, Yifan Zhao, Juan Chen, Zhihong Zhang

**Affiliations:** 1 State Key Laboratory of Digital Medical Engineering, School of Biomedical Engineering, Hainan University, Haikou, 570228 Hainan China; 2 Britton Chance Center and MoE Key Laboratory for Biomedical Photonics, Wuhan National Laboratory for Optoelectronics-Huazhong University of Science and Technology, Wuhan, 430074 Hubei China; 3 Princess Margaret Cancer Centre, University Health Network, 101 College Street, Toronto, Canada

**Keywords:** Breast cancer, Sentinel lymph node, Metastasis, Targeted therapy, Melittin, Nanoparticles

## Abstract

Sentinel lymph node (SLN) metastasis is an important promoter of distant metastasis in breast cancer. Therefore, the timely diagnosis and precise treatment are crucial for patient staging and prognosis. However, the simultaneous diagnosis of metastasis and the implementation of imaging-guided SLN therapy is challenging. Here, we report a melittin-loaded and hyaluronic acid (HA)-conjugated high-density lipoprotein (HDL) mimic phospholipid scaffold nanoparticle (MLT-HA-HPPS), which dually-target to both breast cancer and its SLN and efficiently inhibit SLN metastasis in the LN metastasis model. The melittin peptide was successfully loaded onto HA-HPPS via electrostatic interactions, and MLT-HA-HPPS possesses effective cytotoxicity for breast cancer 4T1 cells. Moreover, the effective delivery of MLT-HA-HPPS from the primary tumor into SLN is monitored by NIR fluorescence imaging, which greatly benefits the prognosis and treatment of metastatic SLNs. After paracancerous administration, MLT-HA-HPPS can efficiently inhibit primary tumor growth with an inhibition rate of 81.3% and 76.5% relative to the PBS-treated control group and HA-HPPS group, respectively. More importantly, MLT-HA-HPPS can effectively inhibit the growth of the metastatic SLNs with an approximately 78.0%, 79.1%, and 64.2% decrease in SLNs weight than those in PBS, HA-HPPS, and melittin-treated mice, respectively. Taken together, the MLT-HA-HPPS may provide an encouraging theranostic of SLN drug delivery strategy to inhibit primary tumor progression and prevent SLN metastasis of breast cancer.

**Graphical Abstract:**

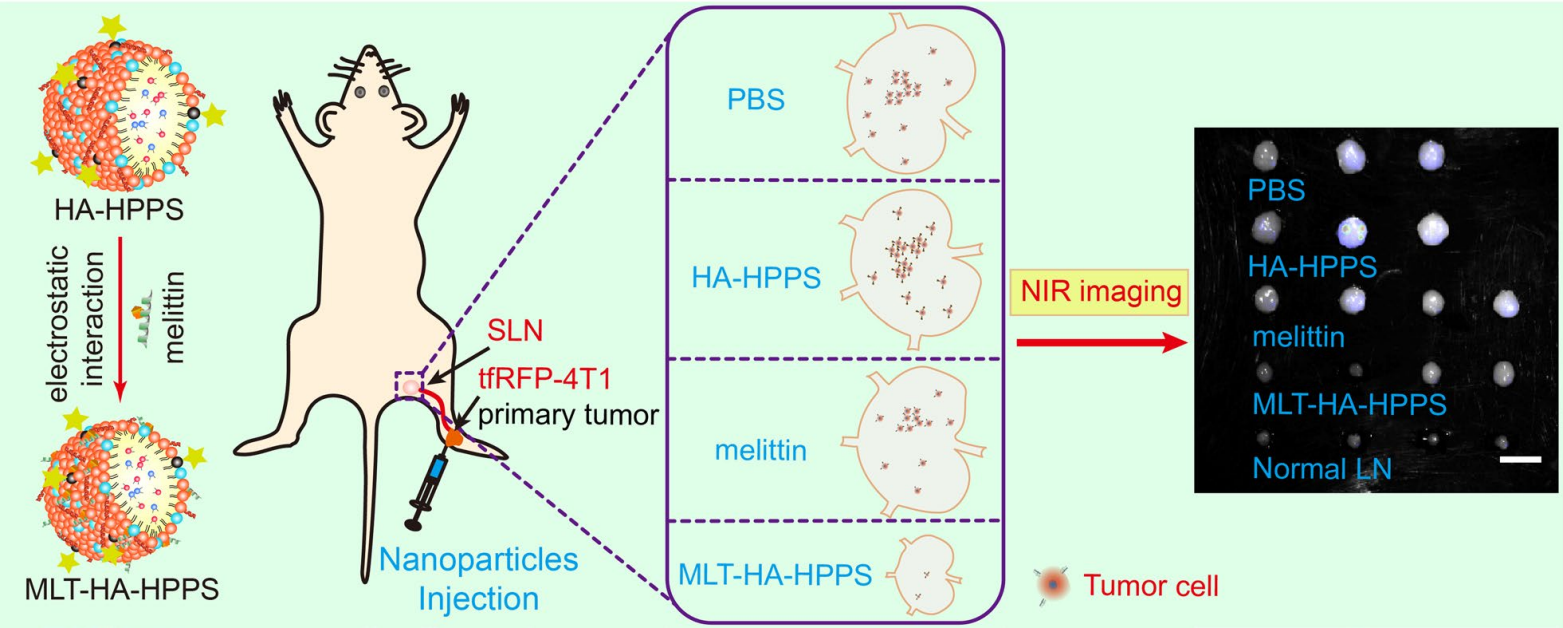

**Supplementary Information:**

The online version contains supplementary material available at 10.1186/s12951-023-02026-7.

## Introduction

According to the latest Global Cancer Statistics 2020 report, female breast cancer has overtaken lung cancer as the most diagnosed cancer, with an estimated 2.3 million new cases, and both incidence and mortality rates are increasing every year [1, 2]. Malignant tumors have become one of the most important public health issues threatening human health, cancer metastasis is responsible for up to 90% of mortality in breast cancer patients, which is extremely difficult to treat in the clinic [3, 4]. The sentinel lymph node (SLN) is typically the first site reached by malignant cancer cells as they spread through the lymphatic system, where most solid tumors can metastasize [5, 6]. Despite significant advances in the current approach to treating primary tumors, it is not easy to deliver therapeutics to metastatic sites, especially to SLN.

Clinical surgery and radiotherapy are currently the most common methods of treating LN metastases [5]. However, due to the challenges of completely removing metastases with conventional surgery, especially the scattered microscopic foci that are easily missed, and the ineffectiveness of conventional chemotherapy delivery to metastatic regions, there exists a possibility for residual tumor tissue to further develop to distant metastases [7, 8]. In addition, the adverse effects of SLN dissection, which include discomfort, lymphedema, and joint dyskinesia, may affect the patient’s quality of life [9]. Therefore, the development of novel methods to efficiently deliver therapeutics to the SLN, where tumors metastasize, could considerably suppress tumor growth and reduce mortality in cancer patients.

Recently, nanoparticles with targeting probes and drug payloads are expected to overcome this problem by efficiently delivering complex drug molecules to tumor sites to inhibit metastatic tumor progression alone or in combination with other therapies [10–14]. Nanoparticles may therefore offer a new avenue for inhibiting tumor SLN metastasis. J. Wang et al. synthesized iCluster/Pt nanoparticles, which incorporate the chemotherapeutic drug cisplatin (Pt) into the polyamidoamine (PAMAM) nanoparticle. After intravenous administration, iCluster/Pt can first naturally aggregate at the tumor site before depolymerizing into smaller PAMAM that can be transferred to the tumor metastatic SLN and inhibit the growth of 4T1 tumor cells [15]. Cabral and co-workers prepared DACHPt nanomicelles by encapsulating oxaliplatin in polymeric micelles. They observed that DACHPt-containing nanomicelles could preferentially accumulate in SLN and reside in tumors after intravenous injection, thereby effectively suppressing LN metastasis and reducing the incidence of tumor recurrence [16]. These studies have highlighted the importance of LN delivery via systemic administration by utilizing the vascular leakage characteristics of tumors. However, it’s frequently required to increase the drug concentration in the SLN by increasing the administered nanodrug dose, because tumor vascularization is difficult in the early stages of metastasis [17]. In addition, many systemic therapies are associated with toxicities and some may even be prometastatic [18, 19]. Therefore, it remains a great difficulty to use system administration nanoparticles to target tumor cells in the early stages of SLN metastases, and it is of great desire to develop an alternative SLN metastasis delivery strategy that is independent of the metastasis tumor SLN vasculature.

It has been reported that epithelial-derived tumors mainly use tumor lymphatics to disseminate from the primary tumor to LNs [20, 21], by which we are inspired to propose that tumor lymphatics could be an alternative route to deliver tracers or nanotherapeutics to LNs. The efficacy of this pathway has recently been validated by intratumoral or paracancerous injection of nanotherapeutics. For example, C. Liang et al. showed that simultaneous laser irradiation of in situ breast cancer tumors and metastatic LN after intratumoral injection of single-walled carbon nanotubes can successfully suppress cancer metastasis to SLN [22]. By encapsulating the chemotherapeutic drug cyclophosphamide in lipid nanoparticles modified with the cell-penetrating peptide R9 for target breast cancer, H. Hu and co-workers improved the inhibitory effect on primary tumor and tumor SLN metastasis via intratumorally injection of the nanomedicine [23]. However, these studies may still have some shortcomings. Firstly, after intratumorally injection, the nanoparticles are mainly taken up by antigen-presenting cells (APCs) and thus alleviate the efficiency to target and eradicate metastatic tumor cells in the SLN. Additionally, these nanoparticles lack an inherent diagnostic capability to accurately determine whether SLN metastasis has occurred. Therefore, there is an urgent need to develop a multifunctional nanoprobe that can efficiently identify the tumor metastatic status of the SLN while also directly targeting and eradicating metastatic cancer cells in the SLN.

**Scheme 1:**
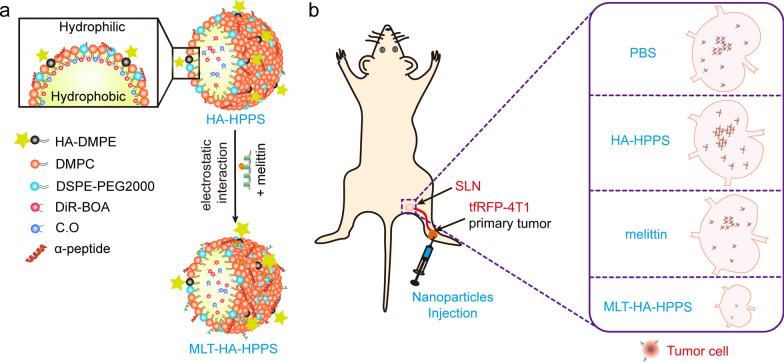
(**a**) Components and structures of the CD44 and SR-B1 dual-targeting HA-HPPS and MLT-HA-HPPS nanoparticles. (**b**) Growth inhibition of breast cancer SLN metastasis by melittin-loaded MLT-HA-HPPS

Melittin, a natural cationic host defense peptide consisting of 26 amino acid residues (GIGAVLKVLTTGLPALISWIKRKRQQ) derived from bee venom, possesses tumor cell cytotoxicity and immunomodulatory functions [24, 25]. With its strong positive charge properties, melittin can interact with biological membranes and disrupt their integrity by forming transmembrane pores, which can directly induce tumor cell apoptosis or necrosis [26]. However, the in vivo applications of this peptide are limited due to the side effect of hemolysis after intravenous injection. Previously, we developed a high-density lipoprotein (HDL)-mimicking peptide-phospholipid scaffold (named HPPS), whose structure could be precisely controlled by an α-helical peptide [27]. Subsequently, we designed a hybrid peptide based on α-helical peptide and successfully loaded melittin onto the scaffold to form a melittin-lipid nanoparticle, which effectively shielded the positive charge of melittin within the phospholipid monolayer, resulting in reduced hemolysis [28–30]. Recently, we developed a CD44 and scavenger receptor class B1 (SR-B1) dual-targeted hyaluronic acid (HA) conjugated HDL mimic phospholipid scaffold nanoparticle (HA-HPPS) with fluorescence/photoacoustic dual-modal imaging capability [31]. Our results confirmed that HA-HPPS effectively targets metastatic 4T1 cells in SLN, and HA-HPPS combined with photoacoustic microscopy imaging can distinguish 4T1 cells metastatic SLN from inflamed and normal LNs. Therefore, in this proof-of-concept study (Scheme 1), we hypothesize that HA-HPPS loaded with the cytolytic melittin peptide, which has a direct tumor killing function, could effectively inhibit the growth of primary breast cancer as well as the metastasis of tumor cells in SLN. In this study, the melittin peptide was successfully loaded onto dual-targeted HA-HPPS nanoparticles via electrostatic interactions, and the melittin loaded HA-HPPS nanoparticles (donated as MLT-HA-HPPS) possess effective cytotoxicity for 4T1 cells. MLT-HA-HPPS exhibit the ability to target tumor SLN and inhibit primary tumor growth and SLN metastasis, thus offering a promising therapeutic approach for the clinical treatment of SLN metastasis in breast cancer.

## Results

### Characterization of MLT-HA-HPPS in vitro

**Fig. 1:**
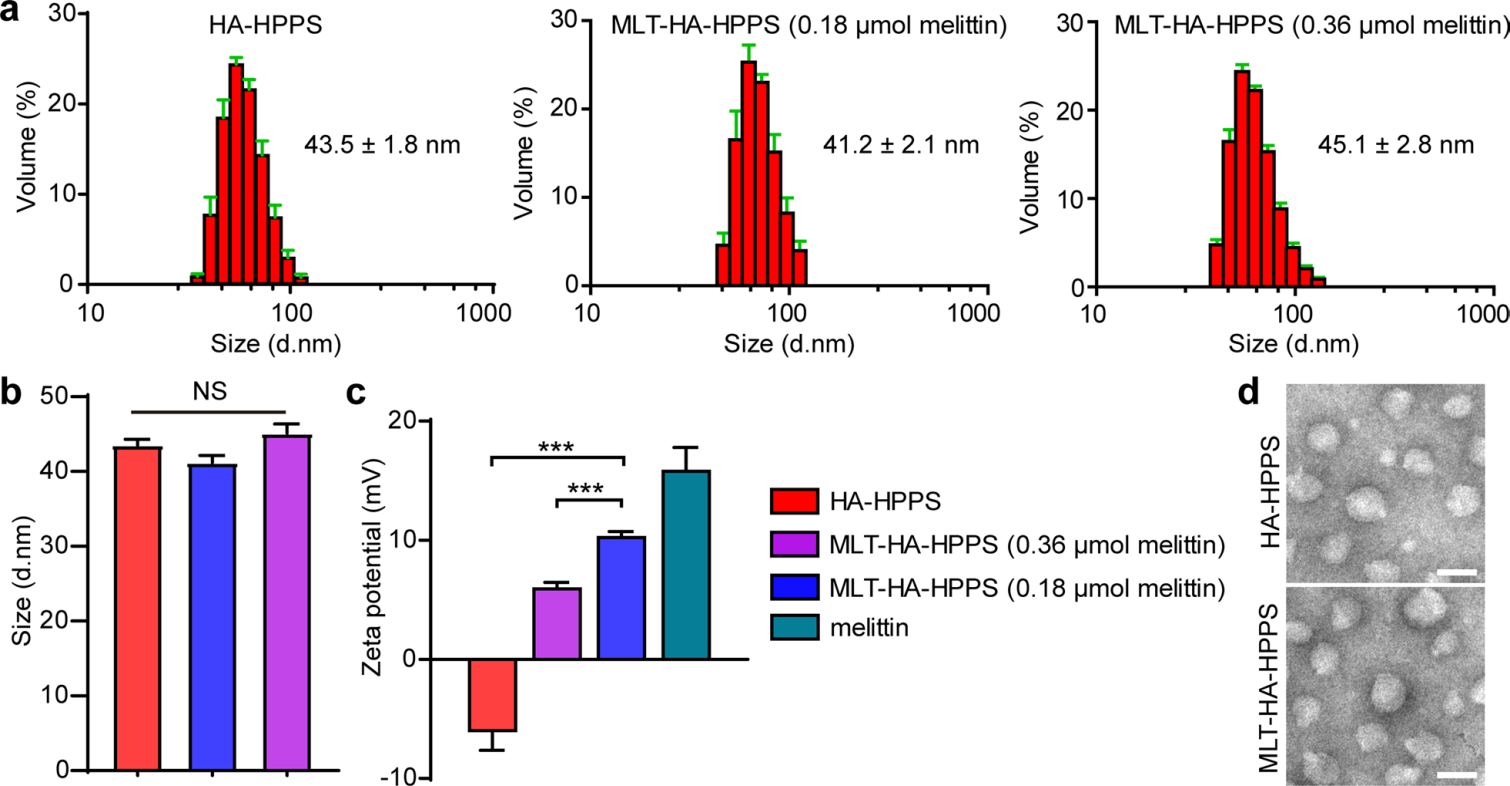
Characterization of dual-targeted nanoparticles. (**a**) The size distribution of different contents (0.18 μmol and 0.36 μmol) of melittin-loaded dual-targeted nanoparticles by using dynamic light scattering (DLS). (**b**) Quantitative analysis of the DLS size data derived from (a). (**c**) The zeta potential of HA-HPPS, free melittin, and MLT-HA-HPPS with different melittin contents tested in water solvent. (**d**) TEM image of HA-HPPS and MLT-HA-HPPS. Data are presented as the mean ± SD, Scale bar: 50 nm. n = 3. ***P < 0.001

Based on our pre-developed dual-targeted HA-HPPS nanoparticles (Scheme 1a) with negative charges, the positively charged cytolytic melittin should be able to bind to HA-HPPS through electrostatic effects. Therefore, to determine the appropriate amount of melittin added to HA-HPPS, we first optimized the amount of melittin for the synthesis of melittin-loaded dual-targeted MLT-HA-HPPS. The method used to synthesize HA-HPPS was reported in our previous study [31]. After the synthesis of HA-HPPS nanoparticles, different amounts of melittin were added to the purified HA-HPPS solution and stored overnight at 4℃. After ultrafiltration, the content of melittin peptide, the size, and zeta potential on MLT-HA-HPPS were examined. The results in Table 1 shown that MLT-HA-HPPS was loaded with 0.076 ± 0.009 μmol and 0.043 ± 0.014 μmol of melittin when adding 0.18 μmol and 0.36 μmol of melittin in HA-HPPS, respectively. This result indicated that the melittin had been successfully loaded onto the HA-HPPS by the electrostatic effect. Dynamic light scattering (DLS) measurement results indicated that the MLT-HA-HPPS showed a size of 41. 2 ± 2.1 nm (0.18 μmol melittin) and 45.1 ± 2.8 nm (0.36 μmol melittin), respectively, which were not significantly different from that of HA-HPPS (43.5 ± 1.8 nm) (Fig. 1a, b). The suitable size (< 100 nm) of the MLT-HA-HPPS should be appropriate for lymphatic delivery [32]. In addition, the zeta potential profiles indicated that HA-HPPS carried a negative charge (− 6.18 ± 1.61 mV) and melittin carried positive charges (15.17 ± 2.52 mV) (Fig. 1c). However, after loading with melittin, the MLT-HA-HPPS presented positive charges, but the charge of 0.18 μmol melittin MLT-HA-HPPS (10.53 ± 0.57 mV) was higher than that of 0.36 μmol melittin MLT-HA-HPPS (6.27 ± 0.41 mV) (Fig. 1d), it can be inferred that the MLT-HA-HPPS with 0.18 μmol melittin bound more of the substance. Transmission electron microscopy (TEM) result clearly showed that HA-HPPS and MLT-HA-HPPS (0.18 μmol melittin) had uniform spherical morphologies and similar particle size (Fig. 1d). To verify the stability of MLT-HA-HPPS, we initially assessed how the size and zeta potential of the MLT-HA-HPPS hydrate changed before and after lyophilization and rehydration. Our results indicated that the MLT-HA-HPPS hydrate could be easily lyophilized and then rehydrated, if necessary, while the size and zeta potential of the MLT-HA-HPPS were not significantly changed (Additional file 1: Fig. S1a, b). In addition, semi-native SDS–polyacrylamide gel electrophoresis (SDS-PAGE) and fluorescence imaging showed that the dual-labeled MLT-HA-HPPS with fluorescein isothiocyanate (FITC) and DiR-BOA was still stable in the 10% FBS and 10% plasma even for 24 h at 37 °C, as both the FITC and DiR-BOA fluorescence signals were detectable in the same band (Additional file 1: Fig. S1c), whereas incubation with 10% Triton-X 100 disrupted the structure of MLT-HA-HPPS and showed unrestricted diffusion of FITC and DiR-BOA fluorescence signals. The UV–Vis absorption spectrum showed that MLT-HA-HPPS and HA-HPPS had obvious enhancement peaks at 280 nm (peptide) and 750 nm (DiR-BOA), respectively, whereas the characteristic absorption peak of MLT-HA-HPPS at 280 nm was slightly stronger than that of HA-HPPS with the same molar content of R4F peptide (Additional file 1: Fig. S2), this may be attributed to the loaded melittin on MLT-HA-HPPS. The peptide release profiles are shown in Additional file 1: Fig. S6, and nearly 45% of the total peptide was released from MLT-HA-HPPS after 96 h of dialysis in PBS solution at room temperature. Meanwhile, about 10% and 54% of free melittin leaked out after 3 h and 24 h of dialysis, respectively. Above all, these data suggested that the melittin was successfully loaded onto MLT-HA-HPPS via electrostatic effects with a size of nearly 40 nm and carrying positive charges.

**Table 1:** Characterization of dual-targeted nanoparticles with different amount of melittin

	R4F (μmol)	MLT (μmol)	Size (d. nm)	Zeta potential (mV)
HA-HPPS	0.298 ± 0.019	**–**	43.5 ± 1.8	− 6.18 ± 1.61
MLT-HA HPPS (0.18 μmol MLT)	**–**	0.076 ± 0.009	41.2 ± 2.1	10.53 ± 0.57
MLT-HA-HPPS (0.36 μmol MLT)	**–**	0.043 ± 0.014	45.1 ± 2.8	6.27 ± 0.41

*MLT* means melittin peptide

### In vitro evaluation of cellular uptake and cytotoxicity of MLT-HA-HPPS to 4T1 cells

**Fig. 2:**
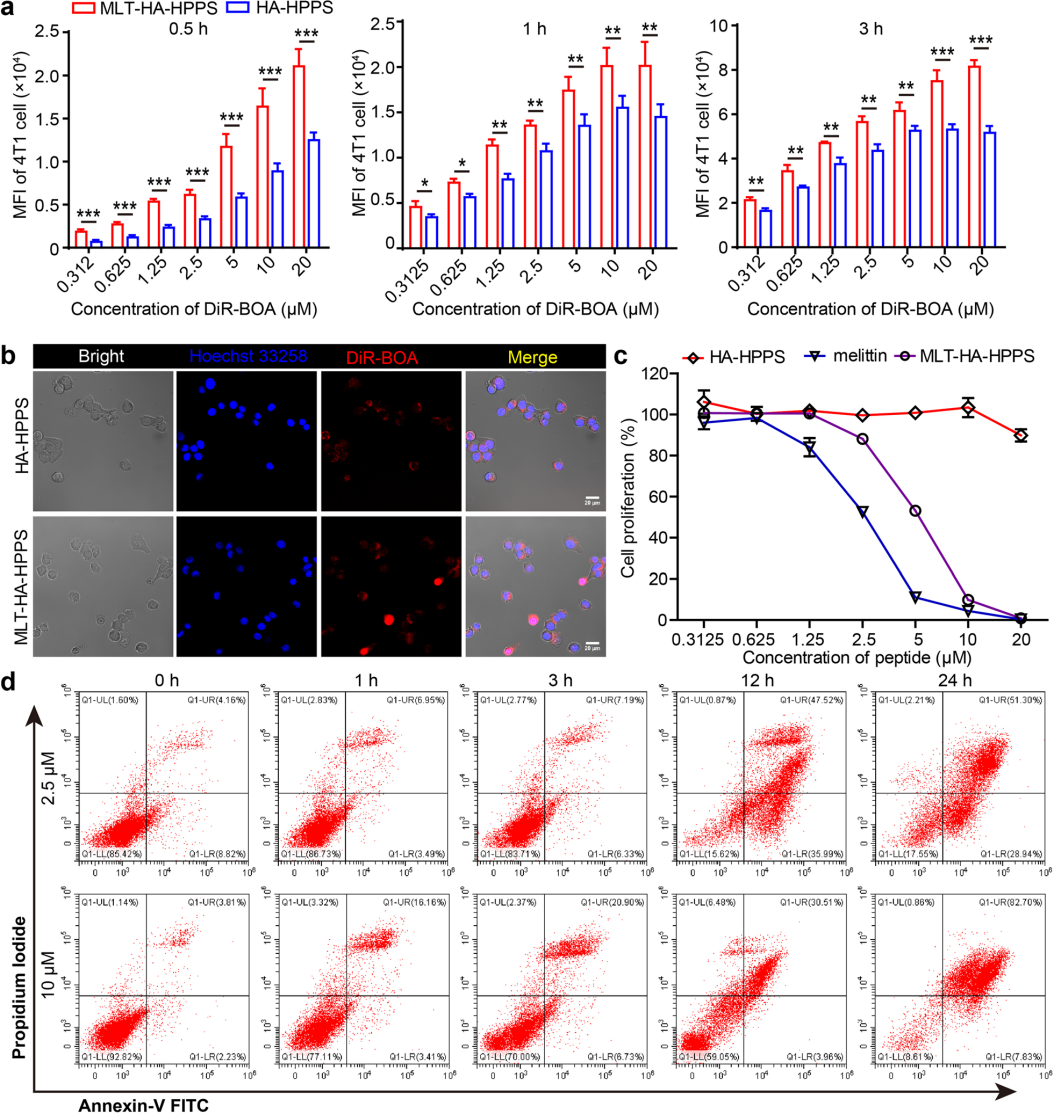
In vitro evaluation of cellular uptake and cytotoxicity of MLT-HA-HPPS to 4T1 cells. (**a**) Flow cytometry (FCM) was performed to analyze and compare uptake of HA-HPPS and MLT-HA-HPPS by 4T1 cells in vitro after exposure to various concentrations for 0.5 h, 1 h, and 3 h. (**b**) Confocal imaging was performed to compare 4T1 cell uptake of HA-HPPS and MLT-HA-HPPS in vitro after exposure to DiR-BOA (10 μM) for 1 h incubation. (**c**) Proliferation assays evaluating the cytotoxicity of melittin, HA-HPPS, and MLT-HA-HPPS on 4T1 cells for 3 h. (**d**) Scatter plot of the FCM assays examining the cytotoxicity of MLT-HA-HPPS in 4T1 cells at different time and concentrations. Data are presented as the mean ± SD. n = 3. **P < 0.01, ***P < 0.001

After synthesizing the melittin-loaded dual-targeted MLT-HA-HPPS, we first verified their ability to target breast cancer 4T1 cells, which highly expressed SR-B1 and CD44 receptors [33, 34]. Nanoparticles with different concentrations of DiR-BOA (0.3125, 0.625, 1.25, 2.5, 5, 10, and 20 μM) were incubated with 4T1 cells for 0.5 h, 1 h, and 3 h, respectively, and the intracellular signal intensity was measured by flow cytometry (FCM) and confocal imaging. The FCM results showed that 4T1 cells took up nanoparticles in a concentration and time-dependent manners, the mean fluorescence intensity (MFI) of DiR-BOA in 4T1 cells incubated with MLT-HA-HPPS was much stronger than that of HA-HPPS, with 1.7 times (0.5 h), 1.5 times (1 h), and 1.6 times (3 h) difference at a high concentration of DiR-BOA (20 μM) (Fig. 2a), respectively. In addition, confocal imaging results also clearly showed that the fluorescence signal intensity in 4T1 cells treated with MLT-HA-HPPS was much stronger than that of HA-HPPS after 1 h of incubation (Fig. 2b). These results indicated that the ability of MLT-HA-HPPS to target 4T1 cells was significantly improved by the addition of melittin, thus providing a suitable candidate for its capacity to destroy tumor cells and prevent their metastasis to LN in vivo. Melittin has a strong ability to kill tumor cells through disrupting the phospholipid bilayer of cell membranes by forming transmembrane pores. Therefore, we further assessed the cytotoxicity of MLT-HA-HPPS in tumor cells by comparing the half-maximal inhibitory concentration (IC_50_) of melittin and MLT-HA-HPPS. The cell survival results showed that within the incubation concentrations, HA-HPPS had essentially no effect on tumor cell survival (Fig. 2c), compared with the IC_50_ of free melittin (2.66 ± 0.09 μM), MLT-HA-HPPS appeared to decrease the cytotoxicity on tumor cells at 3 h, as indicated by an increased IC_50_ value of 5.21 ± 0.13 μM, suggesting that the toxicity of melittin was partially shielded by the phospholipid layer and HA molecules after melittin was loaded onto the dual-targeted nanoparticles. However, when the incubation time of MLT-HA-HPPS with 4T1 cells was prolonged for 6 h and 12 h, the cytotoxicity of MLT-HA-HPPS was stronger than that of free melittin (Additional file 1: Fig. S3), with a decreased IC_50_ of MLT-HA-HPPS (1.21 ± 0.11 and 0.86 ± 0.08 μM) compared to free melittin (2.12 ± 0.15 and 1.38 ± 0.07 μM) for 6 h and 12 h, respectively. The enhanced cytotoxicity of MLT-HA-HPPS for 4T1 cells may be due to the slow release of melittin on MLT-HA-HPPS with a longer incubation time and may also be related to the targeting ability of MLT-HA-HPPS for 4T1 cells. Finally, we investigated the time- and concentration-dependent cytotoxicity of MLT-HA-HPPS against tumor cells and the mechanism underlying cell death. 4T1 cells were treated with 2.5 μM and 10 μM melittin of MLT-HA-HPPS for different times and then stained with Annexin V-FITC/PI for FCM analysis. As shown in Fig. 2d, the percentage of tumor cell death gradually increased with increasing incubation time. Low concentrations (2.5 μM) of MLT-HA-HPPS induced both early apoptosis (lower-right quadrant) and necrosis (upper-right quadrant) in 4T1 cells, while high concentrations (10 μM) of MLT-HA-HPPS primarily resulted in 4T1 cells necrosis (82.7%) (upper-right quadrant). These above results indicated that MLT-HA-HPPS not only exerts an enhanced ability to target 4T1 cells, but also has greater cytotoxicity to induce cell apoptosis and necrosis.

### In vivo tracking of HA-HPPS and MLT-HA-HPPS to target normal popliteal LN

**Fig. 3:**
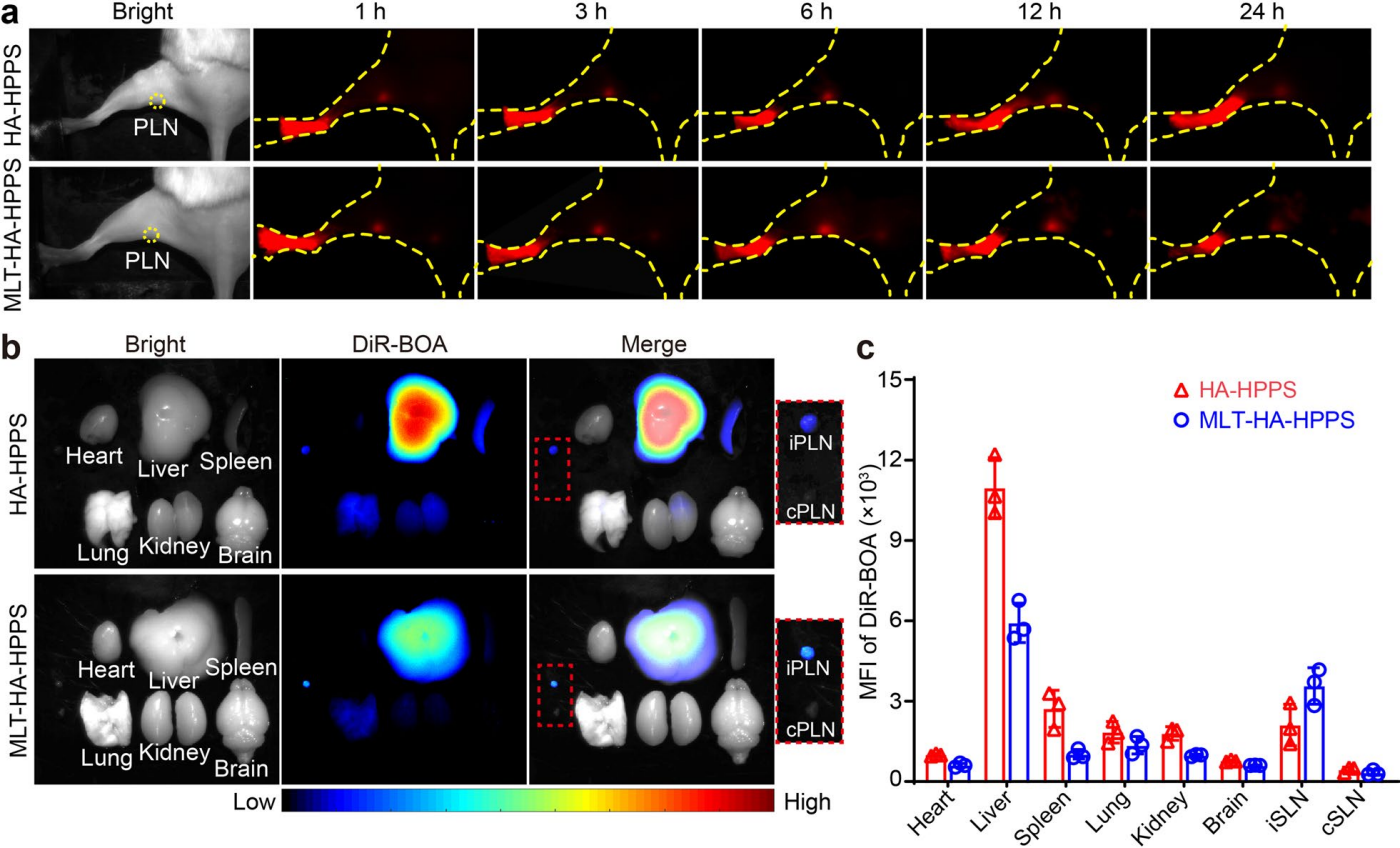
In vivo tracking of HA-HPPS and MLT-HA-HPPS to target normal popliteal LN. **a** Fluorescence imaging of the target ability of HA-HPPS and MLT-HA-HPPS in normal popliteal LNs (PLN) at 1, 3, 6, 12, and 24 h after footpad injection. **b** Representative fluorescence imaging of the dissected organs and LNs after 24 h footpad injection. **c** Quantitative analysis of the MFI of DiR-BOA in the organs and PLNs (n = 3 per group). *iPLN* ipsilateral popliteal LN, *cPLN* contralateral popliteal LN

In our previous study, we demonstrated the target ability of HA-HPPS in normal LNs [31]. Therefore, we speculated that the MLT-HA-HPPS might also be able to target normal LN. To verify this, DiR-BOA labeled MLT-HA-HPPS and HA-HPPS were subcutaneously injected into the footpad of normal mice, and the fluorescence signals in the normal popliteal LN (PLN) were dynamically monitored at 1, 3, 6, 12, and 24 h. As shown in Fig. 3a, the PLNs of the MLT-HA-HPPS group showed a strong DiR-BOA fluorescence signal equal to that of the HA-HPPS group at 1 h, and the fluorescence signals remained detectable up to 24 h. To further confirm the LN targeting ability of MLH-HA-HPPS, we dissected the ipsilateral PLN (iPLN) and contralateral PLN (cPLN) of mice at 24 h post-injection. The in vitro imaging results showed that both the iPLN of the MLT-HA-HPPS and HA-HPPS groups showed stronger fluorescence signals rather than the cPLN (Fig. 3b, c). In addition, in vitro organ fluorescence imaging showed that HA-HPPS was mainly distributed in the liver, spleen, lung, kidney, and iPLN after 24 h footpad injection of nanoparticles, while MLT-HA-HPPS was mainly distributed in the liver, lung, and iPLN (Fig. 3b, c). Furthermore, the immunofluorescence imaging of iPLN showed that the MLT-HA-HPSS and HA-HPPS were mainly taken up by macrophages (F4/80^+^) and dendritic cells (DC) (CD11c^+^) (Additional file 1: Fig. S4). Thus, these results suggested that MLT-HA-HPPS can effectively target LNs after subcutaneous injection. Finally, we tested the in vivo adverse effects of nanoparticles, the functional markers of both the liver (ALT, AST, and TBIL) and kidney (BUN) were distributed in the normal range after 24 h and 48 h nanoparticles injection (Additional file 1: Fig. S5a). Meanwhile, the pathological examination of the major organs (heart, liver, kidney, spleen, lung, brain, and iPLN) by hematoxylin–eosin (H&E) staining suggested that there was no obvious organ damage or tissue denaturation for HA-HPPS and MLT-HA-HPPS treatment compared to normal mouse organs (Additional file 1: Fig. S5b). Thus, the above results suggest that MLT-HA-HPPS can effectively target LNs after subcutaneous injection while maintaining great biosafety.

### In vivo comparisons of the migratory capabilities of HA-HPPS and MLT-HA-HPPS to popliteal SLN

**Fig. 4:**
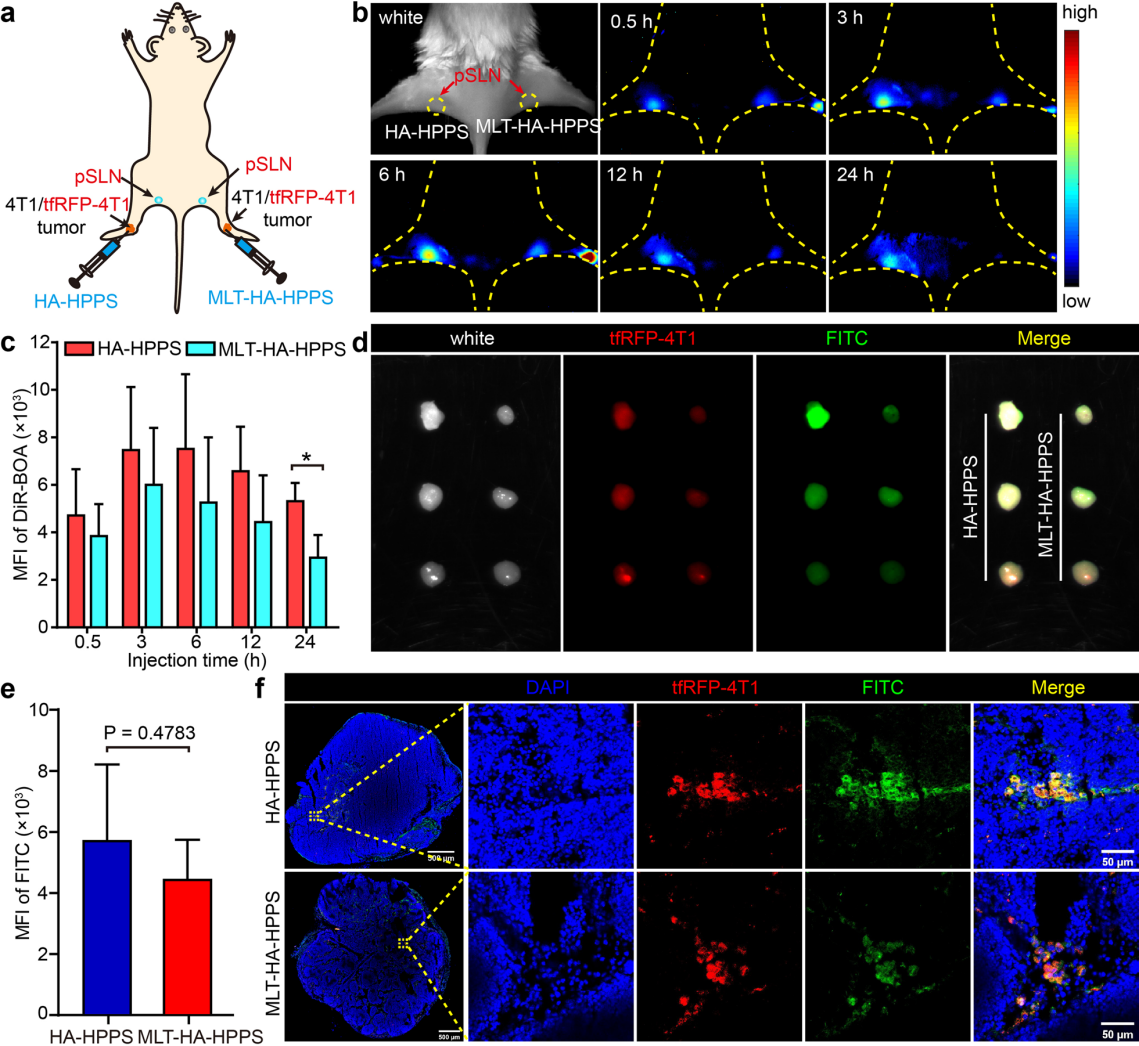
In vivo comparisons of the migratory capabilities of HA-HPPS and MLT-HA-HPPS to popliteal SLN. **a** Schematic of popliteal SLN (pSLN) tumor metastasis detection after six days of 4T1 cells inoculation. **b** Representative fluorescence images to observe the migratory capabilities of HA-HPPS and MLT-HA-HPPS to pSLN at 0.5, 3, 6, 12, and 24 h after paracancer injection. **c** Quantitative analysis of the MFI of DiR-BOA in pSLN. **d** Six days after hock inoculation of tfRFP-4T1 cells (1 × 10^6^ cells/mouse), fluorescence imaging of resected pSLNs were performed at 6 h after paracancer injection of FITC-labelled HA-HPPS and MLT-HA-HPPS. Red: tfRFP-4T1 cells, green: FITC-labelled nanoparticles. **e** Quantitative analysis of the MFI of FITC on resected pSLNs. **f** Confocal microscopy verified the ability of FITC-labelled HA-HPPS and MLT-HA-HPPS to target 4T1 cells in tumor metastasis SLNs. Blue: DAPI, Red: tfRFP-4T1 cells, green: FITC-labelled HA-HPPS and MLT-HA-HPPS. Data are presented as the mean ± SD. n = 3 mice/group. *P < 0.05

After verifying the cytotoxicity of MLT-HA-HPPS against tumor cells in vitro, we next tested the ability of MLT-HA-HPPS to migrate to tumor metastatic SLN and target 4T1 cells in vivo. Six days after inoculation of 4T1 cells into both hocks of BALB/c mice, MLT-HA-HPPS and HA-HPPS were paracancer injected into the hocks area (Fig. 4a), and fluorescence imaging was performed to dynamically monitor the fluorescence signal intensity in the popliteal SLN (pSLN) at different time points. As shown in Fig. 4b, the signals of MLT-HA-HPPS and HA-HPPS could be detected in the pSLN as early as 0.5 h after injection, and the fluorescence signal reached its strongest at 6 h (Fig. 4b). Quantitative analysis of the mean fluorescence intensity (MFI) of pSLN showed that the MFI of pSLN in the HA-HPPS group was greater than that in the MLT-HA-HPPS group, but there was no significant difference between them except at 24 h (Fig. 4c).

Finally, the in vivo abilities of HA-HPPS and MLT-HA-HPPS to target 4T1 cells were examined in a tumor metastatic SLN model, which was established by hock inoculation of a tetrameric far-red fluorescent protein-labelled 4T1 cells (tfRFP-4T1 cells) into the legs of BALB/c mice. Six days after tumor inoculation, FITC-labelled nanoparticles were injected paracancerously into the primary tumors and pSLNs were harvested 6 h later. Fluorescence imaging showed that pSLNs had bright fluorescence signals of both the tfRFP protein and FITC (Fig. 4d), indicating that the tfRFP-4T1 cells metastasized and nanoparticles accumulated in the SLNs. A similar MFI of FITC was observed between MLT-HA-HPPS and HA-HPPS in the quantitative analysis of resected pSLNs (Fig. 4e). Confocal imaging of pSLN sections showed that a large number of tfRFP-4T1 cells appeared in the mice with tumor metastatic SLNs. More importantly, both FITC-labelled HA-HPPS and MLT-HA-HPPS were colocalized with most of the tfRFP-4T1 cells in SLNs (Fig. 4f). These above results indicated that MLT-HA-HPPS could not only efficiently migrate to the pSLN like HA-HPPS, but also effectively target 4T1 cells in vivo, thus providing an effective tool for delivering melittin to the tumor SLN.

### MLT-HA-HPPS effective in inhibiting breast cancer growth in vivo

**Fig. 5:**
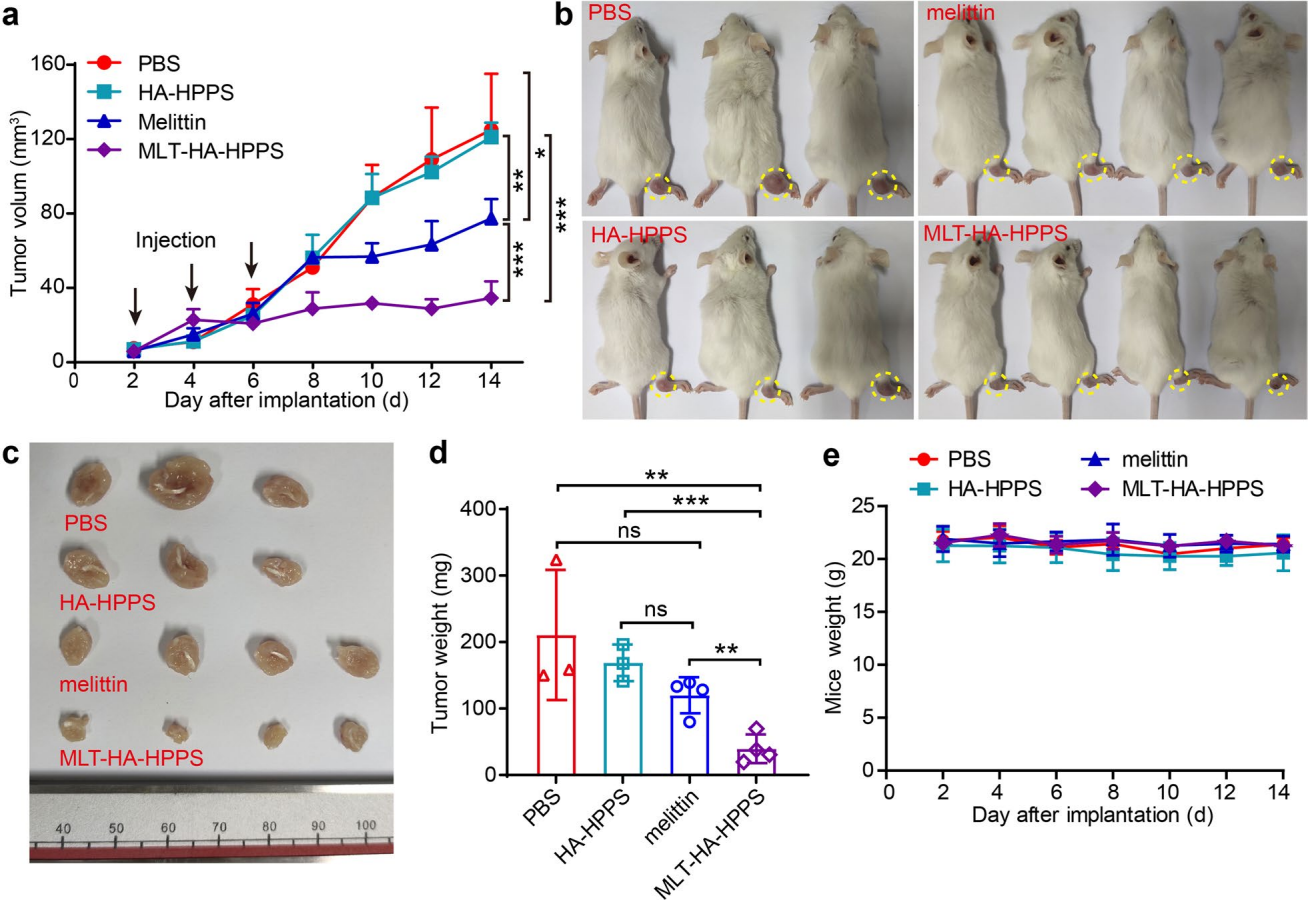
In vivo evaluations of the inhibition effect of MLT-HA-HPPS on the breast cancer growth. **a** Tumor volume curves of 4T1 tumors in each group with three times paracancer injection after 2 days of hock inoculation tfRFP-4T1 cells. Tumor volume in the MLT-HA-HPPS-treated group was significantly inhibited compared to the other groups. **b** In vivo mice photographs and **c** Ex vivo tumors from each group on the 14th day. **d** Quantitative analysis of tumor weight in different groups. 3–4 mice/group. **e** Changes in body weight of mice during treatment. Data are shown as the mean ± SD. ns means not significant. *P < 0.05, **P < 0.01, ***P < 0.001

Having demonstrated the ability of MLT-HA-HPPS to effectively kill tumor cells and target SLN, we next investigated the ability of MLT-HA-HPPS to inhibit breast cancer growth and SLN metastasis in vivo, which is a LN metastasis tumor model established by hock inoculation of tfRFP-4T1 cells into the right legs of BALB/c mice. On days 2, 4, and 6 after tumor inoculation, the mice were treated with paracancerous injections of 20 nmol peptide of HA-HPPS, melittin, and MLT-HA-HPPS in PBS, with a total volume of 40 μL, while equal volume of sterile PBS was injected as a control group. The changes in hock tumor volumes and body weight of the mice were continuously monitored on different days. The tumor growth curve showed that MLT-HA-HPPS dramatically suppressed tumor growth on the right legs (Fig. 5a). Compared with the tumor volume of the PBS group (125.1 ± 30.1 mm^3^), mice treated with HA-HPPS (121.2 ± 7.7 mm^3^) had no effect on tumor growth 14 days after tumor inoculation. Interestingly, mice treated with melittin and MLT-HA-HPPS were able to significantly inhibit the hock tumors growth, while MLT-HA-HPPS (34.5 ± 9.0 mm^3^) showed more excellent ability to suppress tumor growth compared to the melittin group (77.5 ± 10.3 mm^3^). Both the in vivo images of mice (Fig. 5b) and the in vitro image (Fig. 5c) and weights of the resected tumors further confirmed the significantly efficient inhibition of tumor growth by MLT-HA-HPPS (39.6 ± 21.5 mg), with an inhibition rate of 81.3% and 76.5% relative to the PBS-treated control group (210.9 ± 80.0 mg) and HA-HPPS group (168.7 ± 27.5 mg), respectively (Fig. 5d). Although, the weights of tumors in the melittin group (120.1 ± 27.1 mg) were lighter than those in the PBS and HA-HPPS groups, there were no significant difference between them. In addition, it is worth mentioning that there were no differences in the body weight of tumor-bearing mice during the treatment period (Fig. 5e), leading to the preliminary conclusion that MLT-HA-HPPS had no remarkable toxic effects on mice after paracancer administration. These results indicated that the melittin-loaded dual-targeted MLT-HA-HPPS carrying melittin possessed the ability to significantly inhibit breast tumor growth in vivo while maintaining high biocompatibility via paracancer administration.

### Growth inhibition of breast cancer SLN metastasis

**Fig. 6:**
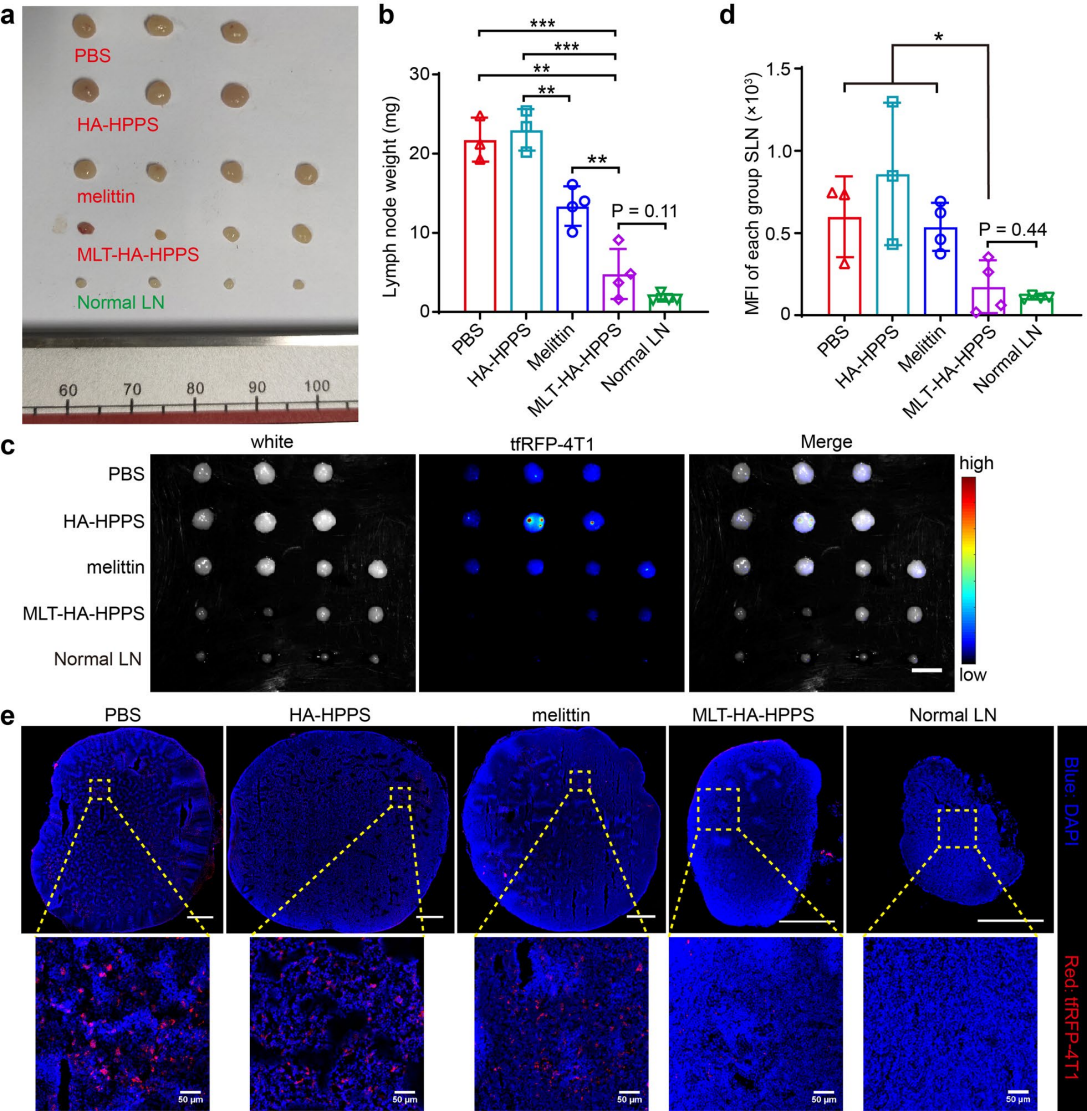
In vivo evaluations of the effect of MLT-HA-HPPS on breast cancer SLN metastasis. **a** Photos of SLN in different groups after 14 days of treatment. **b** Quantitative analysis of SLN weight in different groups. **c**–**d** Ex vivo fluorescence imaging and quantitative analyze the MFI of each group of resected SLNs after 14 days of tumor treatment. Scale bar: 3 mm. **e** Confocal imaging detects tfRFP-4T1 tumor cells in different group of SLNs. Blue: DAPI, Red: tfRFP-4T1. Scale bar: 500 μm. Data are shown as the mean ± SD. *P < 0.05, **P < 0.01, ***P < 0.001

Encouraged by their efficient therapeutic effect on the primary tumor, we finally explored the application of MLT-HA-HPPS in the treating of cancer LN metastasis, taking advantage of its potent LN accumulation capacity. The mice were sacrificed and the metastatic pSLNs were harvested and weighed at the end of the antitumor activity study. The photographs of the resected pSLNs in Fig. 6a showed that the PBS, HA-HPPS, and melittin control groups showed significantly swollen pSLNs, indicating severe lymphatic enlargement due to cancer cell migration and proliferation. In contrast, treatment with MLT-HA-HPPS resulted in the smallest size and weight of pSLNs compared to controls and comparable to normal LNs. Although the average weights of the pSLNs in MLT-HA-HPPS-treated mice (4.8 ± 3.1 mg) were 1.67fold higher than normal LNs (1.8 ± 0.8 mg, *P* = 0.11), they were significantly decreased by 78.0%, 79.1%, and 64.2% than those in PBS treated control mice (21. 8 ± 2.8 mg), HA-HPPS-treated mice (23.0 ± 2.6 mg), and melittin-treated mice (13.4 ± 2.5 mg), respectively (Fig. 6b), indicating that MLT-HA-HPPS effectively inhibited SLN metastasis. Next, ex vivo fluorescence imaging was performed on the harvested pSLN to evaluate the extent of tumor metastasis from different treatment groups. As shown in Fig. 6c, the fluorescence signal of tfRFP-4T1 in the pSLN of the MLT-HA-HPPS group was slightly stronger than the background signal of normal LNs (*P* = 0.44), but considerably weaker than that in the PBS, HA-HPPS, and melittin groups. Attractively, the fluorescence signal of 4T1-tfRFP was barely detectable in half (2/4) of the pSLN in the MLT-HA-HPPS-treated group, which was significantly weaker than that in the other treatment groups. Although the MFI in the melittin group was much weaker than that in the PBS control group and HA-HPPS group, there was no significant difference between them (Fig. 6d). The confocal imaging of SLN sections showed that many tfRFP-4T1 cells could be detected in PBS, HA-HPPS, and melittin groups, whereas the MLT-HA-HPPS treated SLN contained only a limited number of tfRFP-4T1 tumor cells (Fig. 6e). These results suggested that MLT-HA-HPPS can effectively prevent breast cancer cells from metastasizing to SLN in addition to inhibiting the growth of breast cancer in situ. Thus, it offers a potentially valuable therapeutic tool for the diagnosis and treatment of breast cancer LN metastasis.

## Discussion

Metastasis of tumor cells to distal organs is the major cause of death in cancer patients, and epithelial-origin tumors mainly metastasize to distal organs through the lymphatic system, such as breast cancer and melanoma [20, 21]. Tumor cells detached from the tumor in situ first migrate through lymphatic vessels to the SLN and followed metastasize to the distal organs. Surgical resection and radiotherapy are still the main clinical modalities for the treatment of tumor LN metastases. Nanocolloids labeled with the radioactive element technetium (^99^mTc) and blue dye are injected around tumors in the breast to identify the location of SLN, which are then removed and sent for histologic examination. Although this method has proven to be clinically effective, the procedure still has some limitations [35]. In our previous study, we developed a CD44 and SR-B1 dual-targeted fluorescence/photoacoustic nanoprobe HA-HPPS, which can accurately differentiate tumor metastatic SLN from normal LN and inflammatory LN by photoacoustic imaging [31], thus providing an effective tool to identify the metastatic status of tumor SLN. Based on these results, we hypothesize that by carrying tumor-killing drugs, MLT-HA-HPPS not only can be used to identify the metastatic status of tumor SLN, but also effectively inhibit the primary tumor metastasis to SLN. It’s been reported that melittin induces TNBC and HER2-enriched breast cancer death by interfering with growth factor-dependent receptor tyrosine kinase (RTK) interactions critical for receptor phosphorylation and suppressing activation of PI3K/Akt signaling [36, 37]. Therefore, we speculate that the potential suppression mechanism of tumor growth in the present study may be related to the inhibition of RTK and PI3K/Akt pathways.

In this study, we combined melittin peptide with HA-HPPS by electrostatic effect to develop a melittin-loaded dual-targeted MLT-HA-HPPS that has integrated diagnosis and treatment of tumor SLN metastasis. This method of loading melittin by electrostatic effect has the characteristics of simple preparation and operation, in addition, the intra-tumoral/paracancerous injection greatly reduce the amount of injected dose, which can effectively decrease the cost and showed great tumor growth inhibition effect. Our fluorescence imaging results showed that HA-HPPS could effectively label tumor metastatic SLN after paracancerous injection, which is consistent with our previous results [31]. Although the effect of MLT-HA-HPPS in labeling the SLN was slightly weaker than that of HA-HPPS, there was no significant difference within 12 h (Fig. 4b, c). We speculated that the reason for this phenomenon might be related to the positive charge carried on the surface of MLT-HA-HPPS. It has been reported that partially negatively charged nanoparticles have the greatest potential for interstitial transfer to the lymphatic system, because the extracellular matrix of tissues is mainly composed of negatively charged glycosaminoglycans [38]. In contrast, since melittin carries a strong positive charge, the surface of MLT-HA-HPPS shows a positive charge when melittin binds to with negatively charged HA-HPPS (Fig. 1c), which favors accumulation at the injection site and limits its ability to migrate to the SLN.

As a host defense peptide, melittin possesses both direct killing of tumor cells and immunomodulatory functions. However, very low concentrations of melittin can induce hemolytic side effects after intravenous injection, which severely limits in vivo applications. Many efforts have been made to modify the melittin sequence to reduce the hemolysis [39–41]. C. Huang et al. developed an ultra-small size of melittin nanoparticles (α-melittin-NP) [28], α-melittin-NP can not only be effectively shielded the hemolytic properties by the phospholipid layer, but also has a great ability to directly kill melanoma cells, specifically activate liver sinusoidal endothelial cells and APCs in LNs [29, 30]. In this study, we found that MLT-HA-HPPS (IC_50_ = 5.21 ± 0.13 μM) had a weaker ability to kill 4T1 cells compared to free melittin (IC_50_ = 2.66 ± 0.09 μM) (Fig. 2c). We speculated that the strong positive charge of melittin was neutralized by the negatively charged HA-HPPS. In addition, when the melittin interacted with the phospholipid layer of the nanoparticles, which could embed into the phospholipid layer, thus shielding the positive charge of the melittin to reduce the cytotoxicity of the nanoparticles.

Traditional surgical resection of tumors often leads to tumor recurrence and metastasis, and chemotherapy drugs have difficulty reaching microscopic tumor sites. Therefore, the treatment of patients with metastatic tumors is extremely challenging. Currently, researchers have developed various nanodrugs to inhibit primary tumor growth and tumor SLN metastasis with favorable therapeutic effects [42–44]. However, these nanodrugs are mainly taken up by APCs, and it is difficult for the nanodrugs to act directly on the tumor cells, which may have potential toxic effects on the immune cells in the LN. Therefore, a more ideal way for nanoparticles is to be able to act directly on metastatic tumor cells for the treatment of tumor metastatic SLN. Recently, X. Wang et al. prepared a targeted nanoprobe using TMVP1-modified polymer nanomaterials for tumor metastasis SLN imaging and performed imaging-guided photothermal therapy [45]. G. Zhang et al. developed a Cel-loaded erythrocyte-derived microvesicle functionalized with R4F peptide, which showed selective targeting and killing of SR-B1^+^ 4T1 tumor cells and induced T cell-mediated systemic antitumor immune responses in the LN metastatic mode of breast cancer [46]. However, although they all achieved better antitumor effects, dual-targeting melittin nanoparticles may have a more potent antitumor effect than single-targeting nanomedicine strategies. It has been reported that 4T1 cell highly express SR-B1 and CD44 receptors [33, 34]. Our previous study demonstrated that 4T1 cells treated with dual-targeted SR-B1 and CD44 HA-HPPS nanoparticles exhibited enhanced uptake compared to single-targeted nanoparticles [31]. In the current study, we found that dual-targeted SR-B1 and CD44 HA-HPPS and MLT-HA-HPPPS could not only effectively target 4T1 cells in vitro (Fig. 2a, b), but also target 4T1 cells in metastatic SLN in vivo (Fig. 4f). Interestingly, the in vitro results showed that MLT-HA-HPPS carrying melittin had an enhanced targeting effect for 4T1 tumor cells. This may be contribution to the fact that the membrane-disrupting function of melittin and MLT-HA-HPPS carries a positive charge. Since negatively charged phosphatidylserine and O-glycosylated mucin are overexpressed in the plasma membrane of many cancer cells, which results in a slightly higher net negative charge in these cell membranes than in normal eukaryotic cells [47]. Furthermore, our previous results found that the different charges on the surface of tumor cell membranes and APC resulted in the different ability of α-melittin-NP to target and kill these cells [30]. Compared with intravenous injection of nanoparticles, intratumoral or paracancerous injection is more likely to accumulate in the tumor metastasis SLN and minimize the toxic side effects to the organs. Our in vivo therapy results showed that MLT-HA-HPPS treatment significantly inhibited both primary tumor growth and SLN metastasis, while melittin also partially inhibited tumor SLN metastasis (Figs. 5a, 6a), demonstrating the ability of melittin to directly kill tumor cells. In addition, the NIR dye DiR-BOA loaded in the core of the nanoparticles has strong light absorption ability [48], so it is possible to combine photothermal therapy with MLT-HA-HPPS to achieve enhanced inhibition of SLN and distant organs metastasis in the future.

## Conclusions

In conclusion, we developed a CD44 and SR-B1 receptors dual-targeting lipid nanoparticle loaded with melittin, which effectively inhibited primary tumor growth and SLN metastasis. The melittin binds to HA-HPPS via electrostatic effect and its cytotoxicity is successfully shielded within MLT-HA-HPPS, showing excellent suppressed cellular proliferation ability by inducing tumor cell apoptosis and necrosis. Furthermore, our results showed that paracancer injection of MLT-HA-HPPS not only was able to efficiently migrate to the SLN and target the metastatic tumor cells in the SLN, but also significantly inhibited both primary tumor growth and SLN metastasis in vivo. Thus, the above experimental evidence indicated that MLT-HA-HPPS offers a promising therapeutic approach for the clinical treatment of SLN metastasis in breast cancer due to its enhanced tumor cellular uptake and pronounced therapeutic effects.

## Methods

### Materials

1,2-dimyristoyl-sn-glycero-3-phosphoethanolamine (DMPE), 1,2-distearoyl-sn-glycero -3-phosphoethanolamine-N-[methoxy(polyethylene glycol)-2000] (ammonium salt) (DSPE-PEG2000, ammonium salt) and 1,2-Dimyristoyl-sn-glycero-3-phosphocholine (DMPC) were obtained from Avanti Polar Lipids Inc (Alabaster, AL, USA). 5 kDa hyaluronic acids (HA) was obtained from Lifecore Biomedical LLC (MN, USA) (cat#: HA5K-1). Fluorescein isothiocyanate (FITC) was purchased from Sigma-Aldrich Co. (St. Louis, MO, USA). R4F (an ApoA1-mimetic peptide, Ac-FAEKFKEAVKDYFA- KFWD) and melittin peptide (GIGAVLKVLTTGLPALISWIKRKRQQ-NH_2_) were synthesized by Shanghai Apeptide Co., Ltd. (Shanghai, China). The NIR dye DiR-BOA (1,1′-dioctadecyl-3,3,3′,3′-tetramethylindotricarbo-cyanine iodide bis-oleate) was synthesized as previously described [49].

### Mice and cells

BALB/c female mice were purchased from Changsha Hunan Silaike Jingda Laboratory Animal Co., Ltd. (Changsha, Hunan, China). All animal studies were conducted in compliance with protocols that had been approved by the Hubei Provincial Animal Care and Use Committee (2019S2044) and were performed in compliance with the experimental guidelines of the Animal Experimentation Ethics Committee of Huazhong University of Science and Technology. The mouse mammary adenocarcinoma 4T1 cell line was kindly provided by Professor Li Su (Huazhong University of Science and Technology). tfRFP-4T1 cells were obtained by transfecting 4T1 cells with a plasmid containing the tfRFP gene [50]. These cells were cultured in complete RPMI-1640 medium (Gibco, Thermo Fisher Scientific, USA) containing 10% fetal bovine serum (FBS, Gibco) and 1% penicillin–streptomycin (Gibco) in a cell incubator (Thermo, USA) with 5% CO_2_ and 95% air at 37 °C.

### Synthesis and characterization of the HA-HPPS and MLT-HA-HPPS

The HA-DMPE compound and HA-HPPS synthesized as described in our previously study [31]. Briefly, DMPC (3 μmol), DSPE-PEG2000 (0.0114 μmol), cholesterol oleate (C.O, 0.1 μmol), DiR-BOA (0.2 μmol), and HA-DMPE (5kDa, 0.04 μmol HA) in chloroform (400 μL) were dried under a nitrogen stream. Then, 4 mL of PBS solution was added to the dried film and vortexed for 5 min. Subsequently, the mixture was sonicated for ~ 1 h at 48 °C and R4F (0.78 μmol) was added dropwise to the lipid emulsion, and stored overnight at 4 °C. After concentration using centrifugal filter units (30 kDa, Millipore, USA), the HA-HPPS was purified using the Akta FPLC system with a HiLoad 16/70 Superose 6 column (General Electric Healthcare, NY, USA). For the synthesis of MLT-HA-HPPS, the different concentrations of melittin (0.18 μmol and 0.36 μmol) were added to the HA-HPPS nanoparticle and stored at 4 °C overnight, and the MLT-HA-HPPS was purified using ultrafiltration. The peptide concentration was measured using a Pierce Modified Lowry Protein Assay Kit (Thermo Fisher Scientific; Rockford, IL, USA). The size distributions and morphologies of the nanoparticles were measured by DLS on a Zetasizer Nano-ZS90 (Malvern Instruments, Worcestershire, UK) and TEM (TECNAI G2, FEI Company, OR, USA) respectively.

### Confocal imaging and flow cytometry analysis

For confocal imaging, 4T1 cells were seeded (2 × 10^4^ cells/well) into 8-well chambers covering the glass bottoms (Nunc Lab-Tek, Thermo Scientific). The cells were then incubated with HA-HPPS and MLT-HA-HPPS at a DiR-BOA concentration of 10 μM for 1 h, and Hoechst 33258 (0.5 μg mL^−1^) was added 15 min before washing. Fluorescence images were captured using a laser confocal scanning microscope LSM 710 (Zeiss, Germany) with an excitation wavelength of 405 nm for Hoechst 33258 and DAPI, 488 nm for FITC, and 633 nm for DiR-BOA.

For FCM analysis 4T1 cellular uptake test, 4T1 cells were seeded into 24-well plates (5 × 10^4^ cells/well), and HA-HPPS and MLT-HA-HPPS were incubated with the cells at different DiR-BOA concentrations (0.312, 0.625, 1.25, 2.5, 5, 10, and 20 μM) for 0.5 h, 1 h, and 3 h. An Annexin V-FITC/PI apoptosis detection kit (Vazyme Co., Ltd., Nanjing, China) was used to determine the mechanism of MLT-HA-HPPS-induced cell death. Briefly, 4T1 cells were seeded into 24-well plates (5 × 10^4^ cells/well), the cells were harvested after incubation with MLT-HA-HPPS at 2.5 and 10 μM melittin for different time points (1, 3, 12, and 24 h) at 37 ℃, and the cells were stained with Annexin V-FITC/PI solution for 10 min. Both the DiR-BOA and Annexin V-FITC/PI fluorescence signals of the cells were analyzed using a CytoFLEX flow cytometer (Beckman Coulter, Brea, CA, USA). Data were analyzed using FlowJo software (FlowJo, LLC, Ashland, USA).

### Cell proliferation assays

4T1 cells were seeded into 96-well plates (1 × 10^4^ cells/well), and different peptide concentrations (0.312, 0.625, 1.25, 2.5, 5, 10, and 20 μM) of HA-HPPS, free melittin, and MLT-HA-HPPS were incubated with 4T1 cells for 3 h, 6 h, and 12 h at 37 ℃. The cells were washed three times with PBS to remove the drugs and further cultured at 37 °C for 21 h, 18 h, and 12 h, respectively. Cell proliferation assays were performed using CellTiter 96^®^ AQueous One Solution Cell Proliferation Assay (MTS, Promega, G3580).

### Lymph node metastasis model and fluorescence imaging

To establish the LN metastasis model of 4T1 murine breast cancer, 1 × 10^6^ 4T1 cells or tfRFP-4T1 cells in 20 μL PBS were injected into the hock area of BALB/c mice. After six days of cell inoculation, HA-HPPS and MLT-HA-HPPS (DiR-BOA: 20 nmol) were injected paracancerously into the hock area of BALB/c mice, and nanoparticles accumulation in pSLNs were detected and analyzed using a custom-made whole-body optical imaging system at 0.5 h, 3 h, 6 h, 12 h, and 24 h after injection of the probes. Fluorescence images of DiR-BOA were acquired using a NIR filter set (excitation: 716/40 nm; emission: 800/40 nm; exposure time: 20 s). For the in vivo target 4T1 cell assay, fluorescence imaging of resected pSLNs were performed at 6 h after paracancerous injection of FITC-labelled HA-HPPS and MLT-HA-HPPS. Fluorescence images of tfRFP and FITC were acquired with a filter set (excitation: 562/40 nm, emission: 640/40 nm; and excitation: 496/40 nm, emission: 562/40 nm, respectively).

### In vivo SLN metastasis treatment

BALB/c mice with metastatic pSLNs were randomly divided into four groups (3–4 mice/group). Then the different groups were paracancerously injected with PBS (40 μL) or HA-HPPS, melittin, and MLT-HA-HPPS (20 nmol peptide, 40 μL) on the primary tumor after 2 days of tumor inoculation, respectively. The mice were administered the above dose once every 2 days for three times, and their primary tumor volume and body weights were measured. They were sacrificed, and their primary tumors and metastatic SLNs were weighed and collected for fluorescence images and confocal imaging on day 14 after treatment. Primary tumor volume = (length) × (width)^2^/2.

### Statistical analysis

Statistical analysis was performed using GraphPad Prism 8 (GraphPad Software, CA). Significance of the detections and results were determined by one-way ANOVA analysis and Student’s t-test (two tailed) for the in vitro and in vivo studies. Data are presented as the mean ± standard deviation. Significant differences between or among the groups are indicated as follows: ns for no significant difference, * for *P* < 0.05, ** for *P* < 0.01, and *** for *P* < 0.001.

## Supplementary Information

**Additional file 1: Fig. S1** In vitro evaluation of the stability of MLT-HA-HPPS. (a) The photographs of MLT-HA-HPPS hydrate before and after lyophilization and rehydration. (b) The change in size and zeta potential of the MLT-HA-HPPS hydrate and rehydration. (c) SDS-PAGE and fluorescence imaging to evaluate the stability of MLT-HA-HPPS after 12 h and 24 h of incubation at 37 °C. Green: FITC; Red: DiR-BOA. **Fig. S2** UV–vis absorption spectrums of MLT-HA-HPPS, HA-HPPS, and free melittin in PBS solution at room temperature. **Fig. S3** Proliferation assays to evaluate the cytotoxicity of melittin, MLT-HA-HPPS, and HA-HPPS on 4T1 cells after 6 h and 12 h of incubation. Data are presented as the means ± SD, n = 3. **Fig. S4** Confocal imaging verified that the MLT-HA-HPPS and HA-HPPS are mainly taken up by APCs. After footpad injection of nanoparticles for 24 h, the normal PLNs were removed for immunofluorescence imaging. Macrophage: FITC-F4/80^+^, DC: FITC-CD11c^+^. **Fig. S5** Evaluation of the biosafety of nanoparticles in vivo. (a) Biochemical analysis of liver and kidney function of alanine aminotransferase (ALT), aspartate aminotransferase (AST), total bilirubin (T-Bil), and blood urea nitrogen (BUN) (n = 3 per group). (b) Histopathological analysis of H&E-stained organ sections from the hearts, livers, spleens, lungs, kidneys, brains, and iPLNs after 24 h and 48 h of HA-HPPS and MLT-HA-HPPS injection. Scale bar: 50 μm. **Fig. S6** Release profile of peptide from MLT-HA-HPPS using dialysis.

## Data Availability

All data generated or analyzed during this study are included in this published article.
