# Supplementary material for: Effective treatment of metastatic sentinel lymph nodes by dual-targeting melittin nanoparticles
Source: J Nanobiotechnology. 2023 Aug 1;21:245. doi: 10.1186/s12951-023-02026-7 (PMC10391974; doi:10.1186/s12951-023-02026-7)
Supplement: Supplementary file 1 — Additional file 1: Fig. S1 In vitro evaluation of the stability of MLT-HA-HPPS. (a) The photographs of MLT-HA-HPPS hydrate before and after lyophilization and rehydration. (b) The change in size and zeta potential of the MLT-HA-HPPS hydrate and rehydration. (c) SDS-PAGE and fluorescence imaging to evaluate the stability of MLT-HA-HPPS after 12 h and 24 h of incubation at 37 °C. Green: FITC; Red: DiR-BOA. Fig. S2 UV–vis absorption spectrums of MLT-HA-HPPS, HA-HPPS, and free melittin in PBS solution at room temperature. Fig. S3 Proliferation assays to evaluate the cytotoxicity of melittin, MLT-HA-HPPS, and HA-HPPS on 4T1 cells after 6 h and 12 h of incubation. Data are presented as the means ± SD, n = 3. Fig. S4 Confocal imaging verified that the MLT-HA-HPPS and HA-HPPS are mainly taken up by APCs. After footpad injection of nanoparticles for 24 h, the normal PLNs were removed for immunofluorescence imaging. Macrophage: FITC-F4/80+, DC: FITC-CD11c+. Fig. S5 Evaluation of the biosafety of nanoparticles in vivo. (a) Biochemical analysis of liver and kidney function of alanine aminotransferase (ALT), aspartate aminotransferase (AST), total bilirubin (T-Bil), and blood urea nitrogen (BUN) (n = 3 per group). (b) Histopathological analysis of H&E-stained organ sections from the hearts, livers, spleens, lungs, kidneys, brains, and iPLNs after 24 h and 48 h of HA-HPPS and MLT-HA-HPPS injection. Scale bar: 50 μm. Fig. S6 Release profile of peptide from MLT-HA-HPPS using dialysis. [file 12951_2023_2026_MOESM1_ESM.docx]

**Effective Treatment of Metastatic Sentinel Lymph Nodes by**

**Dual-Targeting Melittin Nanoparticles**

Yanfeng Dai^1^, Xiang Yu^1^, Yuehong Leng^2^, Xingzhou Peng^1^, Junjie Wang^2^, Yifan Zhao^2^, Juan Chen^3^, Zhihong Zhang^1,2, *^

^1^ State key laboratory of digital medical engineering, School of Biomedical Engineering, Hainan University.

^2^ Britton Chance Center and MoE Key Laboratory for Biomedical Photonics, Wuhan National Laboratory for Optoelectronics-Huazhong University of Science and Technology, Wuhan, Hubei 430074, China

^3^ Princess Margaret Cancer Centre, University Health Network, 101 College Street, Toronto, Canada

**Supplementary Figures
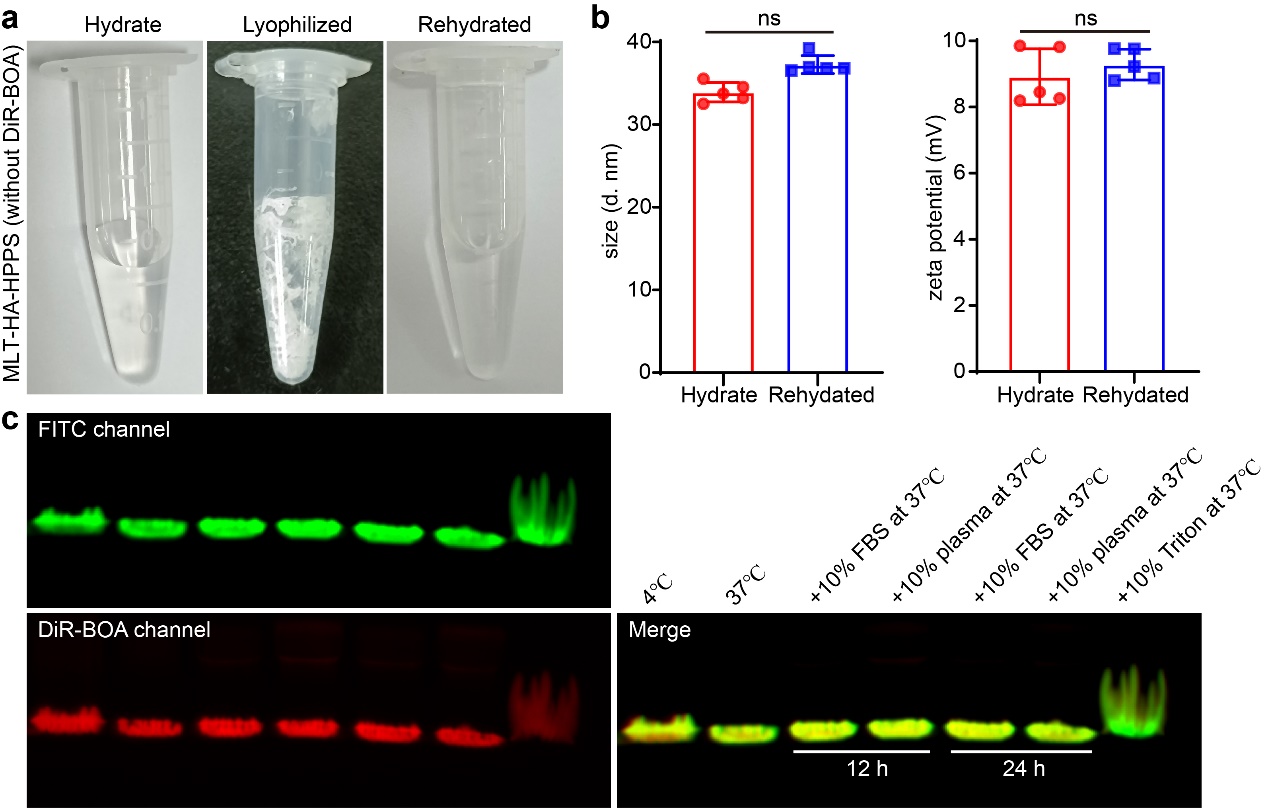
**

**Fig. S1 *In vitro* evaluation of the stability of MLT-HA-HPPS.** (**a**) The photographs of MLT-HA-HPPS hydrate before and after lyophilization and rehydration. (**b**) The change in size and zeta potential of the MLT-HA-HPPS hydrate and rehydration. (**c**) SDS-PAGE and fluorescence imaging to evaluate the stability of MLT-HA-HPPS after 12 h and 24 h of incubation at 37 °C. Green: FITC; Red: DiR-BOA.


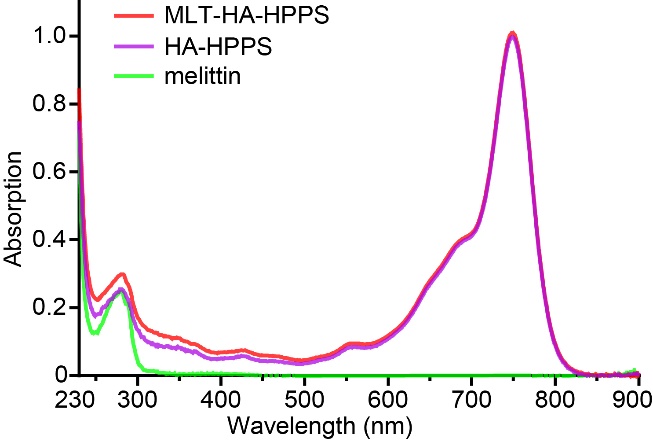


**Fig. S2** UV–vis absorption spectrums of MLT-HA-HPPS, HA-HPPS, and free melittin in PBS solution at room temperature.


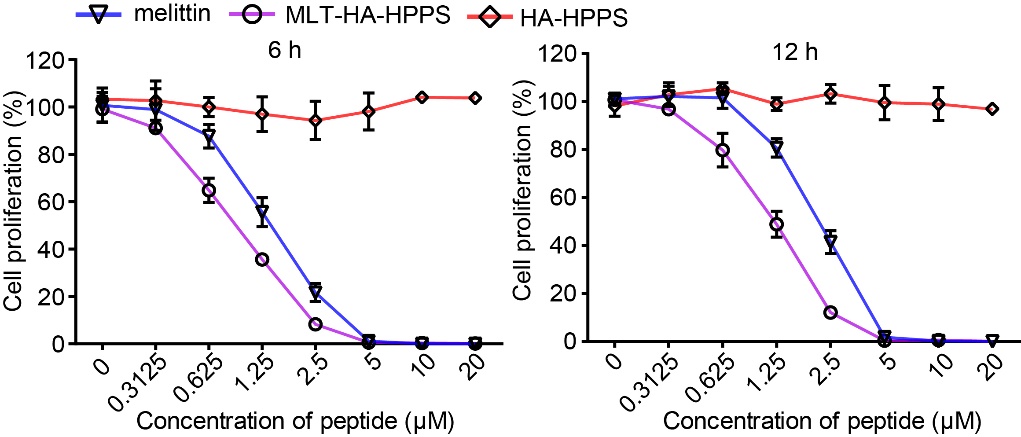
**Fig. S3** Proliferation assays to evaluate the cytotoxicity of melittin, MLT-HA-HPPS, and HA-HPPS on 4T1 cells after 6 h and 12 h of incubation. Data are presented as the means ± SD, n = 3.


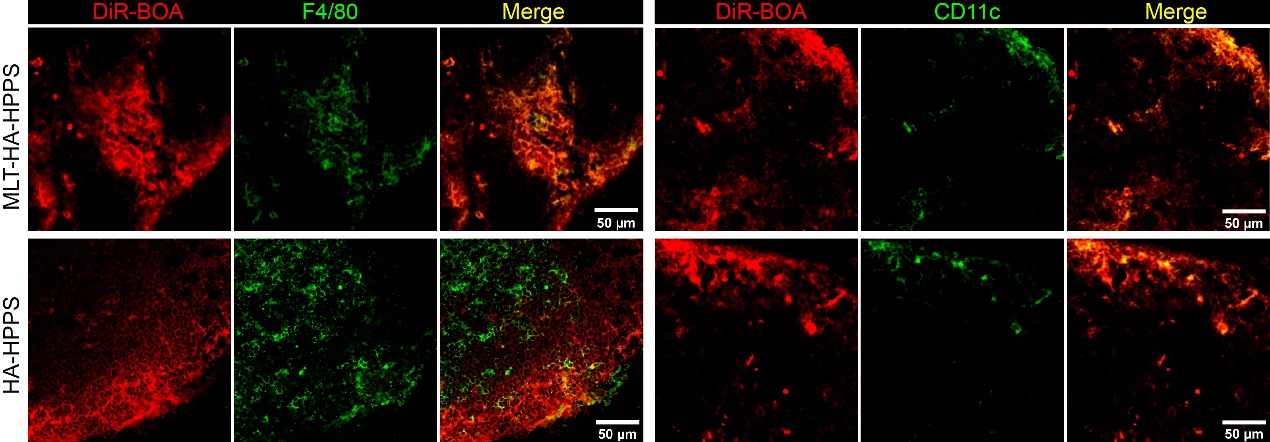
**Fig. S4** Confocal imaging verified that the MLT-HA-HPPS and HA-HPPS are mainly taken up by APCs. After footpad injection of nanoparticles for 24 h, the normal PLNs were removed for immunofluorescence imaging. Macrophage: FITC-F4/80^+^, DC: FITC-CD11c^+^.


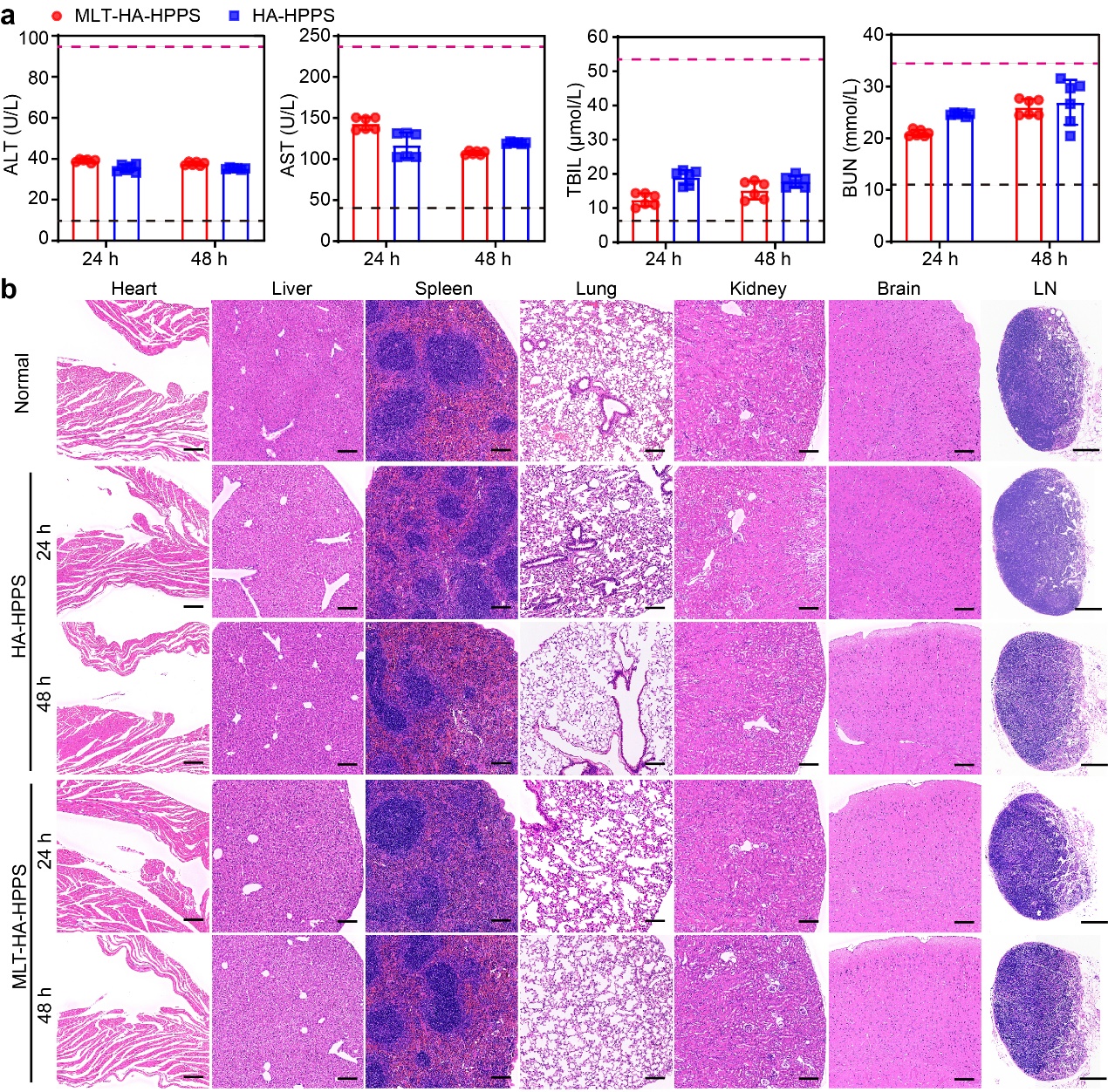
**Fig. S5 Evaluation of the biosafety of nanoparticles *in vivo*.** (a) Biochemical analysis of liver and kidney function of alanine aminotransferase (ALT), aspartate aminotransferase (AST), total bilirubin (T-Bil), and blood urea nitrogen (BUN) (n = 3 per group). (b) Histopathological analysis of H&E-stained organ sections from the hearts, livers, spleens, lungs, kidneys, brains, and iPLNs after 24 h and 48 h of HA-HPPS and MLT-HA-HPPS injection. Scale bar: 50 μm.


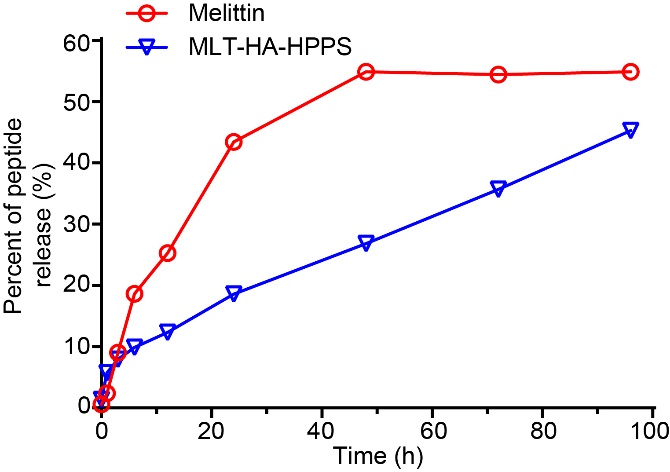
**Fig. S6** Release profile of peptide from MLT-HA-HPPS using dialysis.

Supporting Method

The release of melittin from MLT-HA-HPPS was tested by dialysis. 5 mL of MLT-HA-HPPS solution (11.8 μM/0.0625 mg/mL, peptide concentration) was filled into the dialysis tube (15 kDa). Free melittin with the same concentration was used as a control. MLT-HA-HPPS and free melittin were dialyzed in the phosphate-buffered saline (PBS) at room temperature for 96 h. 50 μL of MLT-HA-HPPS and free melittin solution were taken out from the dialysis tube at 1 h, 3 h, 6 h, 12 h, 24 h, 48 h, 72 h, and 96 h, and the peptide concentrations were measured after the dialysis completed.
